# Lessons learned from including Patient and Public Involvement members throughout research projects in Tic Disorder research

**DOI:** 10.1186/s40900-026-00909-1

**Published:** 2026-06-09

**Authors:** Nikita R. Rattu, Olivia Hastings, Charlotte L. Hall, Kelly-Marie Prentice, Rebecca Woodcock, Emma McNally, Paul Stevenson, Suzanne Parsons, Madeleine J. Groom, Camilla M. Babbage

**Affiliations:** 1 NIHR MindTech MedTech Health Research Centre, School of Medicine, Institute of Mental Health, University of Nottingham, Nottingham, NG7 2TU UK; 2 Nottinghamshire Healthcare NHS Foundation Trust, Nottingham, UK; 3 NIHR Nottingham Biomedical Research Centre, Institute of Mental Health, University of Nottingham School of Medicine, Nottingham, UK; 4 Tourettes Action, The Meads Business Centre, 19 Kingsmead, Farnborough, Hampshire GU14 7SR UK; 5 Genius Within, Scremerston Town Farm Cottages, East Sussex, TD15 2SY UK

**Keywords:** Patient and public involvement and engagement, Health research, Tic disorders, Healthcare

## Abstract

**Background:**

Shared and reflective practice when conducting Patient and Public Involvement and Engagement (PPIE) with underserved communities requires a collaborative approach to understand how best to involve public contributors across the lifecycle of a research project. Researchers from the MindTech research group at the University of Nottingham, UK, conducted three studies aiming to improve healthcare services for people living with tics, and address gaps in current treatment provision: (1) ‘**Tourette’s Hear Us**’ – a qualitative exploration of experiences accessing healthcare for tics; (2) INTEND (**ImproviNg** **Tic** services in **EnglaND**) – a study to develop a care pathway for children and young people with tics and (3) ORBIT-UK (**Online** **Remote** **Behavioural** **Intervention** for **Tics** **UK**) – a study to transform an online behavioural intervention for tics into a digital treatment implemented in services. Each study included a PPIE panel. We present these three case studies of how PPIE was conducted and key learnings across them.

**Methods:**

PPIE panels were actively involved in project design, recruitment, data collection, interpretation, and dissemination. Research teams documented the PPIE activities and their impact on the research. Collaboration between researchers facilitated discussions of the progress and impact of PPIE in each study and enabled shared learning to collectively improve future PPIE methods.

**Learnings:**

Our key learnings, developed through challenge, discussion, and resolution, are presented across the three case studies and include: the importance of representative PPIE panels comprised of members with genuine lived experiences; the provision of safe and inclusive spaces to support members to share their perspectives; supporting communication to facilitate contributions and engagement; tracking and reflecting on the impact of PPIE activities on the project; and sustaining involvement throughout the research cycle, including co-authoring outputs. Evaluating PPIE methods through panel feedback was also highlighted.

**Conclusions:**

This paper provides new insights into how people with tics can be meaningfully engaged as research partners, and additional adjustments that should be made to accommodate the unique and involuntary nature of tics. These learnings extend beyond tic disorders, offering transferable methodologies to strengthen PPIE.

**Supplementary Information:**

The online version contains supplementary material available at 10.1186/s40900-026-00909-1.

## Abstract (plain language summary)

**Background:**

Patient and Public Involvement and Engagement (PPIE) means actively involving people with personal experience of a health condition as collaborators in the research process, not just involving them as study participants. The lack of established healthcare services for people with tics in England has led to long waitlists and insufficient access to specialist treatment. People with tics and their families have described feeling abandoned and let down by healthcare services. It is therefore important that the needs of people affected by tic disorders are prioritised, their voices heard, and their viewpoints considered and acted upon when conducting health research intended to improve outcomes for these groups. Here we describe PPIE in three studies conducted at the University of Nottingham, U.K., focusing on shared learning across the studies and recommendations for how to improve PPIE.

**Methods:**

Across the studies, PPIE members informed the research design and processes, and guided the interpretation of study findings through their lived experience perspectives.

**Learnings:**

We learned how to (1) create genuine, diverse PPIE groups to create comfortable spaces to share lived experiences; (2) design activities for PPIE panel members with diverse needs; (3) maintain contact with PPIE members throughout the research cycle to keep them updated and engaged and seek their feedback.

**Conclusions:**

We aim to continue to improve how PPIE is incorporated into tic disorder research to ensure it is guided by people with lived experience and leads to greater benefits for the tic community.

**Supplementary Information:**

The online version contains supplementary material available at 10.1186/s40900-026-00909-1.

## Background

Patient and Public Involvement and Engagement (PPIE), defined as “*research being carried out‘with’ or ‘by’ members of the public rather than ‘to’*,* ‘about’*,* or ‘for’ them*” by the National Institute for Health and Care Research [1] is crucial to ensuring research is meaningful, relevant, and shaped by lived experience. Increasingly, funding bodies require PPIE as a fundamental component of research design [2], rather than as an optional ‘add-on’ [3, 4]. Involvement guidelines advocate for meaningful stakeholder engagement at every stage of the research process to improve health outcomes, and achieve changes in policy that are likely to lead to benefits for the community [5]. PPIE promotes impact by aligning the research process to lived experience, for example, co-designing effective participant-facing materials, optimising recruitment strategies to support research participation and reduce attrition [6], and guiding the selection of measures to increase the likelihood of meaningful results [7].

Chronic Tic Disorders (CTD) such as Tourette syndrome (TS) affect 1% of the population [8], equating to approximately 110,000 children and young people (CYP) in England alone. Tics are involuntary, sudden repetitive movements (e.g., eye blinking, nose twitching) or vocalisations (e.g., throat clearing, grunting) which typically emerge between the ages of 4 to 7 years. Tics frequently co-occur with other conditions including Attention Deficit Hyperactivity Disorder (ADHD), anxiety, and low mood [9]. People affected by tic disorders face additional challenges, such as pain from carrying out repetitive movements [10, 11] and social stigma [12]. In the U.K. these difficulties are further compounded by limited access to appropriate healthcare, with less than 20% of young people with CTDs having access to evidence-based behavioural therapy [13]. Together, these challenges can severely impact quality of life [14], increase the risk of mental health difficulties including suicidality [15], and contribute to poorer long-term social, educational and financial outcomes [14].

Historically, tic disorder research has focus on reducing tics, with the aim of alleviating impairment. Indeed, health research around tics has previously been criticised for supporting beliefs that tics should be ‘stopped,’ or ‘cured’ [16, 17]. A narrative literature review [18] highlighted the need for research to take a more holistic view, addressing not only tic symptoms but also the wider difficulties of living with tics. The authors also advocated for research and clinical interventions to move away from attempting to ‘cure’ tics and instead towards approaches that strengthen individuals’ resources, resilience, and ability to live well with tics [18]. This underscores the essential role of PPIE in ensuring tic disorder research reflects the priorities of those most affected rather than prioritising medical or research-oriented goals.

Whilst research into other conditions including dementia and autism have long histories of conducting PPIE [19–23], guidance on how to best engage and collaborate with underserved neurodevelopmental research communities, such as those affected by CTDs, remains limited. Furthermore, it is relatively uncommon for neurodevelopmental researchers to publish reports focusing specifically on the process of their participatory methods, despite the potential value of this to others. Publications describing previous research involvement with neurodivergent communities highlighted the value of lived experience contributions in shaping research [24] and made recommendations for how to effectively work with people with lived experience of neurodevelopmental conditions [25–28]. Whilst important, these previous papers lack representation of Tourette syndrome (TS), focusing primarily on autism and ADHD. We hope to address this gap by sharing learnings from working with PPIE members with lived experience of tics and TS specifically.

Research teams from the University of Nottingham have undertaken a range of projects aiming to inform change to healthcare services for people living with tics, and to develop solutions to current inadequacies in treatment provision. These three projects (‘Tourette’s Hear Us’, INTEND and ORBIT-UK) are part of a programme of work delivered by researchers within MindTech, a HealthTech Research Centre for Mental Health at the University of Nottingham.

Early work in this area included ‘**Tourette’s Hear Us**,’ a study that aimed to document and understand the lived experience of accessing healthcare for people with CTDs [29]. Focus groups with adults, young people and parents/carers were carried out to explore experiences of accessing healthcare for tics in the UK. Participants highlighted challenges across the healthcare pathway, including difficulties obtaining a diagnosis, limited access to treatment and a perceived lack of prioritisation of CTDs within services.

The INTEND (**ImproviNg** **Tic** **services** **in** **EnglaND**) study sought to improve access to treatment by developing a care pathway for CYP with tics, supported by a PPIE panel of parents of young people with tics. INTEND found only twelve providers across England with a full care pathway offering both assessment and treatment for CYP with tics [30]. There were also visible regional disparities in tic service provision, with 5 of these 12 full pathway providers based in London.

The ORBIT-UK (**Online** **Remote** **Behavioural** **Intervention** **for** **Tics** **UK**) study was designed to address the impact of the lack of healthcare professionals trained in therapies for tics. The national charity Tourettes Action list only seven NHS behavioural therapists trained to treat tics in young people in the UK [31]. ORBIT-UK, an ongoing study ending in 2027 and informed by a young person and adult PPIE group, aims to transform an online behavioural treatment, found to be effective in the UK [32], to be implemented within the NHS. This provision could enable accessible treatment for young people living with tics who are currently unable to access care.

Each project embedded PPIE throughout, with dedicated groups of adults, young people or parents/carers with lived experience contributing. We present these projects as case studies to highlight practical methods to support meaningful PPIE that has a genuine impact on the outcomes of the study, from inviting individuals to join PPIE groups, engaging, and collaborating with PPIE members, supporting data collection and analysis, and co-authoring outputs. In line with the GRIPP2 checklist [33], we describe how PPIE informed each project and we outline lessons learned to guide future research in tic disorders. This manuscript has been co-authored by researchers and PPIE members from across the three projects.

We will provide an overview of each project, including study aims and methods, report PPIE activities completed within each project and how these activities impacted the research. We will then present and discuss the learnings from across the three projects that we believe enhanced involvement, to support future integration into projects.

## About us

The following studies have been conducted in association with MindTech, a National Institute for Health and Care Research-funded (NIHR) Health Tech Research Centre, focusing on the development and implementation of technology within mental healthcare, and the Institute of Mental Health, a research institute at the University of Nottingham in partnership with Nottinghamshire Healthcare NHS Foundation Trust. We note that none of the academic researchers within the group have lived experience of tics, although our lived experience co-authors have either experience of living with or supporting people with tics (see author positionality statements in Supplementary File 1). Furthermore, each study team included at least one lived experience co-applicant on the respective research grant. For further information about each study and associated PPIE activities, please see Table 1. Both Tourette’s Hear Us and INTEND were co-developed with the https://institutemh.org.uk/research/national-steering-group-for-tics-tourette-syndrome/about-us National Steering Group for tics and Tourette Syndrome, comprising 60 stakeholders across academic, healthcare, education and people with lived experience backgrounds. 

**Table 1:** Overview of PPIE activities for each case study

Aim/s	Study Methods	PPIE Characteristics	PPIE activities
Tourette’s Hear Us			
To explore experiences of children, young people, adults, and parents/carers accessing healthcare for CTDs in the UK (click here for study webpage). To disseminate the findings to stakeholders in the UK.	Two phases: (1) qualitative focus groups with individuals with CTDs and carers; (2) co-development of an animation for an awareness campaign based on findings.	Four White British members (2 male, 2 female), aged 26–59 years. Lived experience included adults with TS, parents of CYP with tics/TS, a research psychologist, researcher in TS in higher education, and third sector involvement (as national TS charity lead and staff member of a CIC).	Co-developed aims; Co-designed recruitment strategy, supported recruitment. Co-designed participant-facing materials, focus group method, and topic guide questions. Facilitated focus group discussions; supported data interpretation and co-authored outputs.
INTEND: ImproviNg Tic services in EnglaND			
To develop a clinical care pathway for CYP with tics for implementation in healthcare services across England (click here for the study webpage).	Four work packages: (1) mapping tic service provision across NHS organisations; (2) exploration of healthcare professionals’ experiences; (3) expert consensus survey to define key pathway components; (4) pathway and guideline development.	Ten parents/carers of CYP with tics/TS (8 female, 2 male; predominantly White British with one Asian British parent), recruited from 10 different regions of England. Panel chaired by EM, national TS charity lead.	Research design developed in collaboration with the National Steering Group for Tics and Tourette Syndrome. Co-designed features of the care pathway. Co-designed dissemination materials. Informed a further funding application.
ORBIT-UK			
To increase access to evidence-based behavioural therapy for CYP with tics by developing ORBIT as an NHS-ready digital intervention (click here for the study webpage).	Single-cohort feasibility study: 20 CYP (aged 9–17) and supporters complete a 10-week, coach-guided online ERP-based intervention.	Two PPIE panels: (1) 10 adults with lived experience (8 female, 2 male), predominantly White British (7) with 2 Indian British and 1 Pakistani British. ; (2) 5 CYP aged 7–16 (age range for young people: 12–17 years) with lived experience of tics (predominantly male).	Co-produced lay summary, informed study aims. Contributed to intervention development, study materials, and user testing. Involved in interview panels and dissemination events

## Impact of PPIE on each project

### Tourette’s Hear Us

PPIE co-designed the aims and analysis, leading to a more meaningful and rich interpretation of the study findings, and increasing their relevance to people with CTDs.

Co-design of participant facing documents made them more understandable to a lay audience and supported recruitment. A PPIE co-investigator facilitated the focus groups, asking the main questions and guiding the discussions, with technical and admin support provided by researcher team members as co-facilitators. This approach helped to develop rapport with focus group members and address power imbalances that some participants might feel between themselves and a research team with no lived experience, leading to improved trust and more empathic and inclusive group discussions.

PPIE members co-produced the animation video with Woven Ink through an iterative feedback process with the team, ensuring it portrayed the lived experience of focus group participants. By co-producing an animation that the team and PPIE members were proud of, members felt motivated to disseminate work they believed in, leading to greater impact and engagement of the animation with non-academic audiences.

### INTEND

A lived experience member (EM), who has faced similar struggles to the panel members, chaired the PPIE panel. This helped guide panel conversations and created more open discussion. The panel’s lived experience aligned with and provided context to study findings. Their views and contributions shaped the design of the recommended care pathway [34], for example, by emphasising the role and benefits of psychoeducation for tics, and by refining the criteria for referral into the pathway. The PPIE panel felt strongly that ‘tics occurring for a duration of at least 12 months,’ was an essential criterion, and that ‘psychological/physical distress or harm,’ and ‘functional impairment’ should also exist as a separate referral criterion. They also stipulated that any of these criteria should be sufficient to warrant a referral. At the time, these criteria proposed by the panel did not align with study findings from the consensus survey used to identify fundamental features of the INTEND pathway. Their feedback prompted a necessary discussion within the study team (including clinical experts) when finalising referral criteria, ultimately leading to a decision to implement these adjustments within the pathway design. Therefore, the INTEND PPIE panel had a crucial, positive impact on the study and the pathway design.

### ORBIT-UK

PPIE members contributions to the reviewing of participant-facing documents such as participant information sheets and research protocols ensured that the documents were clear and relevant to families, and that the tone of the information felt balanced between being approachable and informative. PPIE members involvement in data collection methods including reviewing the participant interview guides and selecting outcome measures allowed them to be shaped by lived experience priorities. The PPIE panel played a large role in co-designing and user-testing the ORBIT-UK platform, including sharing their thoughts on layout, language, and accessibility features. They identified usability issues and suggested improvements to navigation and content, leading to a platform that we expect to be more suitable and acceptable to families. We expect PPIE involvement in this area will lead to more meaningful feedback during the feasibility study relating to, e.g., the content of the platform, rather than technical problems.

Finally, PPIE members’ role in shaping dissemination, which has included features in the bi-monthly ORBIT-UK newsletter, attending events (e.g. the ORBIT-UK Commissioners Event [35]), and co-authorship of conference materials ensures that the dissemination remains relevant to lived experience communities. PPIE impacts dissemination by helping to improve trust with the public that the work has been carried out with people who are living with the condition, enhancing the relevance of the work.

## Learnings across the three projects

**Fig. 1:**
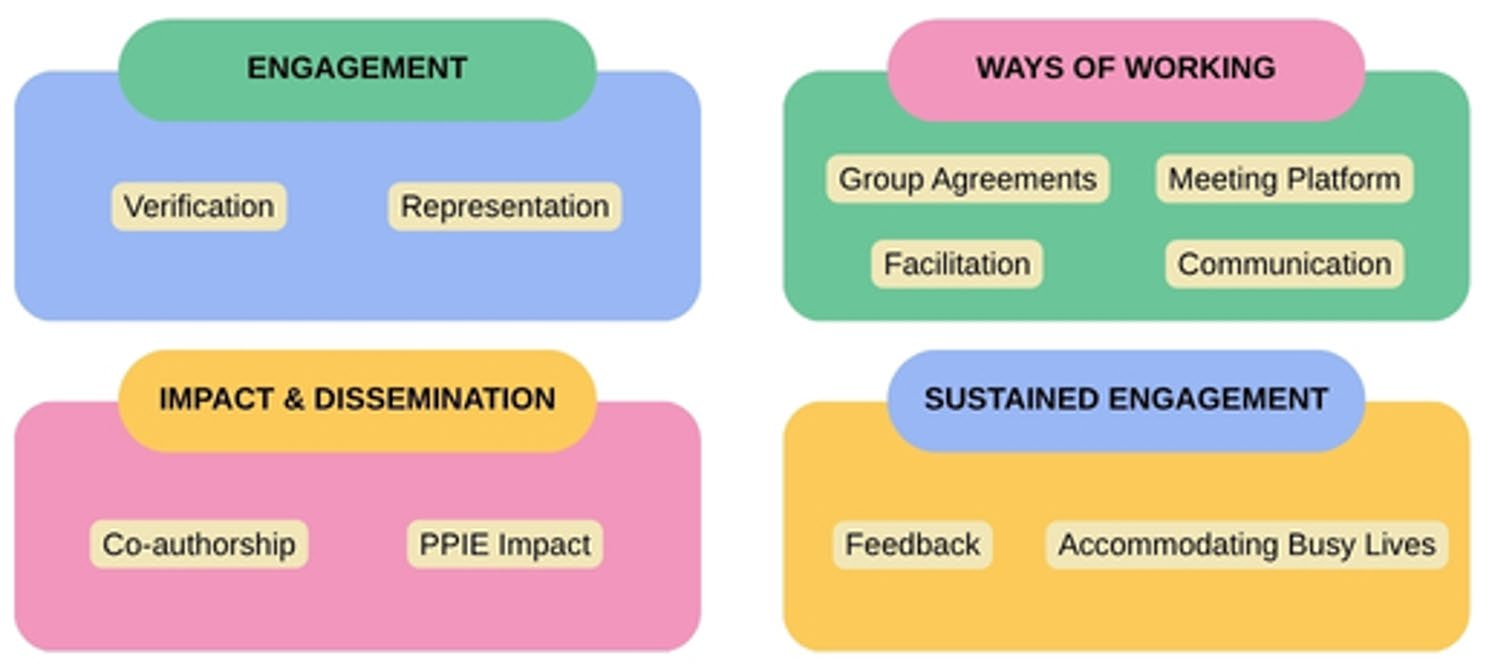
Overview of learnings

Working with different PPIE groups across the three projects within the same institution allowed for reflections across a variety of experiences to support accessible, inclusive and engaging PPIE activities. The two senior authors on this manuscript (CB and MG) worked jointly on the Tourette’s Hear Us project, shaping ways of working with PPIE into future projects, with ORBIT-UK PPIE led by CB and INTEND led by MG. Recognising that the approaches offered insights into PPIE in tic disorder research, a more formalised sharing of learning led to the collaboration of an abstract and poster for presentation at the annual European Society for the Study of Tourette Syndrome 2025. Upon identifying common ideas and learnings that could be applied across different projects, this progressed from organic conversations to regular online meetings. In these meetings, researchers could share, learn from and update one another on activities and how to continue improving collaboration with PPIE members within tic disorder research. Here, we present a summary of the learnings from this collaboration, structured into specific categories including Engagement, Ways of Working, Impact and Dissemination and Sustained Engagement, shown in Fig. 1.

### Engagement

#### Verification

Effective verification is the first step to ensuring that PPIE groups are inclusive, representative and safe for all members. Both the ORBIT-UK and INTEND projects faced challenges when inviting people to work with us.

One challenge involved non-genuine sign-ups to take part in PPIE whereby, during the early PPIE group formation, it became evident that ineligible members had registered. In this context, we refer to individuals that did not have lived experience of tics and were not a parent or carer of a young person with tics. This issue is becoming increasingly common during data collection with participants [36]. In the context of our research, this was likely due to advertising PPIE opportunities online to reach the lived experience community nationally. People without lived experience may have seen these online adverts and signed up disingenuously.

To protect the integrity of the PPIE panel and ensure a safe space for authentic lived experience, the team implemented verification steps such as conducting individual phone calls prior to formal onboarding and requesting postal addresses to ensure PPIE members lived in the UK. These procedures allowed researchers to verify identities while also facilitating introductory conversations to gain an initial understanding of participants’ lived experiences with tic disorders. Current guidelines for how to avoid non-genuine participation predominantly refer to recruiting participants for research studies, rather than inviting PPIE members to work with research teams [37]. Specific verification guidelines for PPIE would be welcomed to ensure efforts towards maximising the representation of marginalised groups within PPIE work are not harmed by restrictive advertising to minimise non-genuine volunteers.

#### Representation

Recognising the diversity within the tic community, such as individuals with ongoing struggles, complex comorbidities, or limited access to services, these projects have deliberately sought to work with individuals whose experiences may be underrepresented in research. Online invitation methods to join PPIE groups have been particularly valuable in this regard, increasing accessibility and allowing for wider geographical representation. This also created opportunities to engage those considered “seldom heard” in PPIE. Rather than labelling these individuals as “hard to reach”, it is crucial that researchers adapt their strategies to reduce barriers to participation [38]. This includes ensuring those with ‘living experience’ of tics, who may have ongoing struggles feel comfortable sharing and ensures all voices are heard. PPIE groups should reflect the full spectrum of experiences, rather than just those who may be ‘higher functioning’, and therefore more likely to be able to take part in research projects.

### Ways of working

#### Group agreements

To support group cohesion and productive meetings, during PPIE panel induction, the researchers developed a ‘Ways of Working’ activity to ensure all members of the PPIE panel could share how they wanted the group to look and feel. Such activities can help set ground rules with consideration of the needs and characteristics of the specific group. Annual reviews ensure the group agreement is updated and relevant throughout the project. A fundamental aspect to consider was PPIE members ticcing during meetings and how to ensure they felt comfortable to do so while meetings proceeded. While common research meeting expectations might include not interrupting others, a PPIE member from the ORBIT-UK panel shared that this might be difficult for them and even trigger their tics. Therefore, during ORBIT-UK PPIE meetings, the team ensured that PPIE panel members knew that they could tic freely and could opt to have videos and microphones on or off during meetings. Across panels, we have learned that giving PPIE members ‘space’ to tic freely allowed them to become comfortable within the meeting environment, encouraging their engagement with activities.

#### Facilitation in meetings

PPIE meetings chaired or facilitated by those with lived experience enabled discussions to be guided by someone who is both a member of the research team and with insight from lived experience perspectives. This provided PPIE members with someone they felt comfortable sharing their thoughts and experiences with and could relate to, both during and post-meetings. It also furthered rapport building and engagement, leading to more meaningful and rich findings. While the research team remained available and continued to conduct roles within their research capacity e.g., through sharing research updates or giving technical and practical support, sharing the roles between the research team and PPIE group helped to overcome the power imbalances often inherent within research [38], improving trust.

PPIE leads were appointed through existing links from previous collaborative working and research. Training was not provided, as PPIE leads already had expertise in PPIE activities. For example, the PPIE lead for the INTEND study is the Chief Executive Officer of Tourettes Action and therefore duties such as chairing meetings, contacting parents and supporting them to share their experiences already existed within their role. Including PPIE leads as co-applicants on grant applications ensured their time was funded fairly, transparently, and in a systematic manner.

#### Meeting platform

The use of online platforms to hold meetings supported increased attendance and allowed for national representation of lived experience members across England, and sometimes UK-wide. In addition, the use of online meetings enabled members to remain involved even when they may be experiencing an increased severity in their tics, as they can opt to turn cameras or microphones on or off during meetings.

#### Supporting communication and involvement

It has been crucial to develop efficient, effective ways of communicating to increase the options for PPIE members to share their input both during meetings and when corresponding between meetings. For example, recognising that members sometimes found it difficult to share their views during meetings, the INTEND team ensured that the order of ‘hand raising’ was followed, so members could indicate that they wished to speak. Throughout projects, facilitators have encouraged the use of the meeting chat if microphones were unavailable, or they did not wish to speak. In addition, if members preferred to share their thoughts privately, they were encouraged to send the team an email with their feedback post-meeting. Overall, ensuring members could contribute in their preferred way enhanced input and allowed all members’ views to be considered.

Outside of meetings, feedback from PPIE groups suggested that large amounts of information being shared with them was sometimes overwhelming. To streamline communication, the teams found that sharing less information but more often was best, for example discussing just one task per email. Within ORBIT’s PPIE panel, members felt this maximised their participation without causing fatigue. Communication with PPIE members after meetings also allowed for continuous collection of meaningful feedback from PPIE members at their discretion. This was particularly useful when capturing views of PPIE members who were unable to attend an online meeting but still wished to review study materials and provide feedback. PPIE members also wanted to share via email updates their own journeys of accessing healthcare for tics and contacting local organisations to raise awareness about the lack of local health services for tics. Therefore, this additional communication between meetings helped to improve collaborative partnerships by ensuring PPIE members who were absent from a meeting felt heard.

### Sustained engagement

#### Feedback

The ORBIT-UK team collected anonymous feedback via Microsoft Forms from members after every session to provide members with regular opportunities to share their views of how PPIE activities were organised, whether positive or negative, and to continually improve their PPIE activities. This may have helped sustain involvement and engagement with the project.

#### Accommodating PPIE members’ availability

As the various PPIE groups consisted of adults, parents, and young people, it was vital to organise activities around their other commitments. To arrange convenient meetings, online polls were used to identify PPIE members’ availability. The research teams recognised that most members preferred evening meetings and therefore adjusted their working hours to suit PPIE members. Furthermore, PPIE members were encouraged to join meetings in ways most suitable for them, from wherever they were, on any device that worked for them.

In the inevitable situation when a PPIE member was unable to attend a meeting, a summary of the meeting was shared. Sharing such resources in multiple, accessible formats, such as a Word document and a meeting recording was useful. This ensured that PPIE members remained updated on study progress and still had an opportunity to share their views. Project teams also recognised the importance of giving members a considerate amount of time to review documents and return feedback, ensuring at least seven days for any activities and providing frequent reminders of feedback deadlines. Financial compensation was given to members following NIHR guidelines [39] and adjusted when activities required more time and effort and when members required additional accommodations.

Accommodating PPIE members’ busy lives allowed for sustained interest and engagement with activities, meaning all members feel seen and heard even when unable to join meetings.

### Impact and dissemination

#### Co-authorship

Dissemination within research, the wide-spread sharing of study findings, is integral to bringing about change and ensuring the research reaches those it will most benefit. A key method is creating materials that can be shared about a study, such as academic papers, videos, and infographics. PPIE at this stage can be a powerful method to share research findings with lived experience communities and beyond. Dissemination typically happens toward the later stages of a project. However, planning ahead ensures challenges such as time commitment for continued involvement in the publication process (perhaps past the end of the research funding period) and anticipating multiple rejections or rounds of review from academic journals to be considered. Ensuring appropriate remuneration and being transparent about potentially lengthy timelines for publication were important. Part of this process must include confirmation from PPIE members that they are happy to be named on public-facing materials or offering alternatives for members who wish to remain anonymous such as acknowledging them within a group, or using a pseudonym. In the projects, PPIE members reported that they valued being invited to contribute to publications as co-authors to edit and provide input, and having their contributions formally acknowledged in outputs.

#### PPIE impact

Alongside this, it is important to monitor the impact of PPIE on research, to determine the extent to which the research aims and objectives are met and how PPIE has contributed to this. The INTEND and ORBIT-UK team used the Public Involvement in Research Impact Toolkit (PIRIT) tracking tool [40] to systematically track the PPIE panel’s input throughout the study. Tourette’s Hear Us used tracking documents to decipher key decisions and progress within the research that PPIE impacted, making it easier to report PPIE activities within publications.

## Discussion

Working with PPIE groups within tic disorder research has provided us with important insights into how to best embed meaningful involvement. We documented and now share these learnings to support other researchers in the area who are developing their experience of working with these groups. Furthermore, our learnings (see Fig. 1) may also facilitate working with PPIE groups with lived experience of other neurodevelopmental conditions. Exploring lived experience of accessing healthcare for tics (‘Tourette’s Hear Us’); developing a care pathway for CYP with tics (INTEND) and implementing an online intervention for tics within the NHS (ORBIT-UK) all share the central aim of improving healthcare provision and outcomes for people with tics. The lived experience voice of our PPIE groups has been integral to every stage of the research process.

Given the unique and involuntary nature of tics, it is essential to consider this when developing guidelines and understanding expectations when working with people with lived experience to create comfortable, positive, collaborative environments. Enhancing PPIE activities to make them suitable, inclusive and engaging for a particular group with lived experiences requires a flexible approach. This involves considering relevant factors such as accessibility, relations and power dynamics between researchers/healthcare professionals and PPIE groups, specification and expectation of roles within PPIE and sufficient compensation and resources [38]. Previous research has also recommended that research teams exercise inclusive practices and remain sensitive to unintentional discrimination [41]. PPIE members should have abundant support when contributing to research and equal opportunities to contribute with adjustments made to accommodate their needs. We believe our work demonstrates learnings in line with these recommendations. For example, we created safe environments where PPIE members felt comfortable to tic freely in meetings, allowed extra time for review tasks, adjusted financial compensation accordingly, and established group agreements to clarify roles and expectations. Furthermore, we ensured that PPIE members had various means to communicate and provide input, including via meeting chat or sending feedback after meetings via email. While this also ensured that each member had equal opportunities to contribute based on their preferences, we found that this allowed them to privately share more about their personal experiences. Therefore, these strategies helped foster trust, inclusion, and meaningful collaboration.

## Limitations and future directions

We have refined our skills for working with PPIE members through finding shared solutions to challenges, and by working collaboratively across projects to enhance shared knowledge and approaches. We hope the learnings shared here will support future research involving people with lived experience of tics and facilitate a smooth, efficient process from the beginning.

While we have documented useful lessons learned to date, we note limitations to our PPIE methods and recognise ongoing improvements are still required. One of these focuses on representation and we identified a number of ways in which we can improve this in future PPIE. Firstly, Tourette’s Hear Us had a relatively small PPIE group with no young people represented, and across our PPIE groups ethnicity was relatively homogenous to white ethnic groups. Due to the heavy resource load required for PPIE, the panel size of groups is often limited by practicalities such as cost and time limitations, as was the case for Tourette’s Hear Us. To ensure suitable representation, whilst recognising having a fully representative group is never possible, ORBIT-UK and INTEND secured a higher level of funding and costed for larger PPIE groups (i.e., around 10 members) allowing for suitable idea generation, changes in membership during the study, and broader representation of ethnicity and age. Future studies should cost appropriately for PPIE resources to ensure that there are sufficient means to work with a sizeable, representative PPIE group early on in a project.

Secondly, our learnings highlight that research teams should determine how to engage with minoritised communities at an early stage of project design. This includes ensuring community groups are aware of health research and PPIE in advance of the project starting and not made to feel they are being approached as an afterthought, or only because of their ethnicity. This would broaden potential PPIE member pools from the outset and allow for non-biased yet diverse PPIE groups. In addition, including positive action statements, where individuals from certain groups are encouraged to engage when inviting members of the public to join PPIE groups may also reduce discomfort around selection. Further increasing representation by improving ethnic diversity and involvement of other underserved groups within PPIE groups in health research, including younger populations, and how to optimise working with these groups should be explored [38, 42].

Third, ensuring a PPIE panel represents people with a variety of needs and lived experiences extends to identifying and monitoring these additional needs, ensuring accessibility adjustments are recognised and implemented from the start.

A further point speaks to our use of online involvement methods which allowed us to substantially increase geographical representativeness, yet there remains a risk of digital exclusion for a small minority of individuals who may not have access to technology, internet, or are not confident using digital devices. Evidence from our prior work suggests that online provision for young people with tic disorders does not appear to systematically exclude participants [43] and smartphone access within this age group is high, over 80% of adults also own smartphones and usage within older age groups is increasing [44]. Future PPIE work should nonetheless adopt flexible, multi-modal engagement strategies, including telephone options. Further, where feasible, in-person options, alongside reimbursement for network connectivity costs should be available to minimise barriers and maximise inclusivity.

Other implications of our work include the importance of fostering sustainable engagement of PPIE members and recognising the needs of the researchers who are supporting them. Given the long timeline of some research, it is crucial to continue exploring how PPIE activities can be sustainable and engaging, encouraging the same members to remain active throughout the lifecycle of research. This will allow PPIE members to make informed contributions to a project, equipped with their own learnings and evolved insights from working on the same project. One approach to sustaining PPIE members’ engagement is to gather feedback throughout the project and ensure they know that their feedback is valued. While the ORBIT team did collect feedback following every PPIE meeting from members, this was not consistently done by the Tourette’s Hear Us or INTEND teams. Therefore, when conducting future tic disorder research, systematically recording feedback around the design, conduct and process of PPIE activities is essential to substantiate any related further learnings. We must also consider that members may decide to withdraw from PPIE work. Participation is not a ‘fixed’ process and can be unpredictable [45]. We should instead focus on valuing each member’s input, for however long this may be, ensuring that PPIE is a mutual learning process for researchers and members, where everyone involved can gain something from their experience.

Conducting research sensitively also involves recognising the different roles, needs, and identities within a research team, including whether researchers themselves have lived experience, and the implications this may carry, which could extend to power imbalances between researchers and PPIE groups [38, 41]. Our approach to mitigate this was having a team member with lived experience facilitate meetings and follow up with PPIE members afterwards. Nonetheless, the PPIE lead for the INTEND study was the CEO of a charity which may have introduced a power imbalance of its own. Such circumstances require careful thought from researchers and PPIE leads to consider and address power imbalances from various viewpoints. We felt the CEO, having faced similar struggles to the PPIE members, helped to reduce possible hierarchies and positioned them more equally in the group, supporting equal and meaningful dialogue. 

One method that can increase PPIE members’ confidence and ability to respond during PPIE meetings is through offering training to members. Within our projects, upskilling PPIE on research methodology or analysis is carried out by the research team which can be resource intensive and challenging to provide. We hope to see increased opportunities for PPIE to receive training in such areas, which we believe will impact their ability to give feedback to the research.

Additionally, while we have mostly reported how to improve the experiences of PPIE members when working on health research on tics, it is also fundamental to consider the needs of researchers. Navigating the complexities of conducting PPIE requires considerable input on behalf of the researcher. Recent articles have started to outline some of the costs, not limited to, but including time, altered working hours, administrative burdens and emotional toll [46]. We will continue efforts within our institution to highlight the value, time and costs of PPIE work, and to provide wellbeing support to peers who are working in PPIE.

The process of discussing and combining learnings across the three projects developed naturally from organic conversations to formal meetings. To ensure a reliable, standardised approach for future PPIE, we suggest working towards a structured approach to logging methods that optimise PPIE within tic disorder research.

## Conclusions

This paper has presented three case studies of PPIE in tic disorder research and reflected on the methodological insights gained. Collectively, our learnings across the three case studies improves understanding of how people with real lived experience of tics can participate as PPIE members to advocate for the needs of people with tics within research. Our experience demonstrates that meaningful involvement requires more than tokenistic consultation: it depends on early integration, ongoing support and communication, and intentional strategies to ensure accessibility and inclusivity. By embedding lived experience at every stage of the research lifecycle, our research outputs more accurately reflect the experiences of people with tics and their families. Longer-term tracking of research impact is needed to understand whether these approaches result in meaningful changes in healthcare for those affected by tics.

Using multi-modal methods, including online methods, we can reach and work with these members, making adjustments that prioritise their needs, preferences and the specific nature of tics. As researchers, we will continue to consider how to reduce barriers and power differences where possible, ensuring that PPIE members feel that they are genuine research partners. We must continue to work to ensure that a diverse range of lived experience is better represented, including those whose experiences may be affected by intersectional factors such as ethnic minority group status. Importantly, these lessons are not limited to tic disorders and may provide transferable methodologies for embedding PPIE in neurodevelopmental research more broadly. This involves navigating barriers such as organising inclusive, effective PPIE activities within the constraints of a research budget [41]. Nonetheless, we hope that these learnings inform other researchers’ work within the field, contributing to more valid, greater quality research that is conducted together *with* the lived experience community, leading to more relevant improvements in healthcare.

## Supplementary Information

Below is the link to the electronic supplementary material.

Supplementary Material 1: Author Positionality Statements

## Data Availability

No datasets were generated or analysed during the current study.

## Abbreviations

ADHD: Attention-Deficit Hyperactivity Disorder
ASD: Autism Spectrum Disorder
CTD: Chronic Tic Disorder
CYP: Children and young people
ERP: Exposure with Response Prevention
INTEND: ImproviNg Tic services in EnglaND
NHS: National Health Service
ORBIT: Online Remote Behavioural Intervention for Tics
PPIE: Patient and Public Involvement and Engagement
TS: Tourette syndrome
UK: United Kingdom
