# Supplementary material for: Lessons learned from including Patient and Public Involvement members throughout research projects in Tic Disorder research
Source: Res Involv Engagem. 2026 Jun 9;12:84. doi: 10.1186/s40900-026-00909-1 (PMC13251301; doi:10.1186/s40900-026-00909-1)
Supplement: Supplementary file 1 — Supplementary Material 1: Author Positionality Statements [file 40900_2026_909_MOESM1_ESM.docx]

# **Supplementary File 1**

# **Author Positionality statements**

**Dr Camilla Babbage** is a White British cis-gender female working at a university as a Research Fellow. CB has been working professionally with children, young people and families with tics for 10 years but does not have personal experience of living with or caring for someone with tics. CB often takes on professional roles that include centring the lived experience in the research being carried out and therefore recognises that this will impact how she interprets and understands her research.

**Olivia Hastings** is a White British female academic research assistant, supporting projects working with young people with neurodevelopmental conditions, mainly Tourette Syndrome and the ORBIT project. OH is passionate that the lived experience voice is embedded in research, and though she does not have tics herself she seeks to ensure lived experiences voices have an impact on the research she does.

**Dr Charlotte Hall** is a White British cis-gender academic researcher specialising in digital mental health research. She does not have Tourette syndrome but leads work on the ORBIT programme. The author acknowledges how professional status and institutional positioning can shape research decisions and seeks to address this through shared decision-making and reflexive practice.

**Emma McNally** is a White British woman and the CEO of the national charity Tourettes Action. She is the mother of a child with Tourette syndrome and has been campaigning since 2020 to improve healthcare provisions for people with Tourette’s. Emma joined Tourettes Action as CEO in January 2022, following sustained advocacy to strengthen services, including leading a government petition after her son was left without care when a local hospital closed its Tourette’s service.

**Kelly‑Marie Prentice** is a white British, female Senior Project and Implementation Manager working in health research and innovation. She does not have lived experience of Tourette syndrome and contributed to the study from a professional and implementation-focused perspective. The author recognises how professional status and institutional positioning can influence research processes and worked with the team to address this through reflexive discussion and shared decision-making with PPIE contributors.

**Nikita Rattu** is a British Indian female Research Assistant. She does not have lived experience of tics but has been working in tic disorder research for two years. Due to the often misunderstood and unique nature of tics and Tourette syndrome, she recognises the importance and essential need for input from the lived experience community to be accurately reflected and implemented within research.

**Paul Stevenson** is a male lived experience contributor with a late diagnosis of Tourette Syndrome, alongside ADHD, OCD, and sensory processing differences. Paul’s perspective is shaped by navigating much of his life without a diagnosis, followed by an understanding of the impact that diagnosis, language, and support can have. He brings insight into how Tourette Syndrome is experienced beyond awareness, particularly in relation to stigma, misconceptions, and real-world barriers in education, employment, and public life. Paul also highlights the influence of clinical language at diagnosis, where framing can affect identity, confidence, and access to support. Through his role in advocacy and public engagement, he contributes a lived experience perspective that sits alongside academic and clinical expertise, with a focus on improving understanding and shaping more effective, person-centred support.

**Suzanne Parsons** is a White British cis-gender female she does not have Tourette syndrome herself. She is the mother of two children with Tourette syndrome. Suzanne brings 27 years of lived experience caring for and supporting individuals with the condition. Since 2016, Suzanne has also been actively involved in the Tourette’s community as a Support Group Coordinator with the national charity Tourettes Action, facilitating in person support groups and delivering psychoeducation sessions to academic and healthcare professionals. With a background in psychological research, Suzanne recognises the importance of collaborative working between researchers, developers, individuals with Tourette syndrome and carers with lived experience. She advocates that integrating these perspectives is key to developing accessible, practical, individual, and family focused interventions. In 2022, Suzanne joined Tourettes Action as the Support Services Manager.

**Professor Maddie Groom** is a White British cis-gender female. She does not have lived experience of Tourette Syndrome but has observed the impact of neurodiversity (including tics) in her family. She leads research into healthcare service pathways for chronic tic disorders in children and young people and the development of digital technologies to support care for neurodevelopmental conditions. Maddie recognises the influence of her role as a senior academic on stakeholder engagement and discussions with the lived experience community. She monitors and evaluates this regularly through ongoing reflection, adapting her approach to changing contexts and relationships.

**Rebecca Woodcock** is a White British cis-gender female. She does not have lived experience of Tourette Syndrome but has observed the impact of neurodiversity (including tics) in her family. She leads patient and public involvement at MindTech and her role includes embedding lived experience across mental health and/or neurodiversity research. She recognises that despite her own lived experience of neurodiversity and mental health conditions, her professional status can influence her perspectives and seeks to address inequitable power dynamics (evident and less evident) within both research processes and how we include lived experience contributors.
